# Exposure to secondhand smoke in health institutions and sources of knowledge: a cross-sectional study from the city of Bulawayo, Zimbabwe

**DOI:** 10.11604/pamj.2020.35.46.15341

**Published:** 2020-02-18

**Authors:** Nkanyiso Ndlovu, Mokoko Percy Kekana, Sogo France Matlala, Thembelihle Sam Ntuli

**Affiliations:** 1 University of Limpopo, Department of Public Health, Private Bag X1106, Sovenga, 0727, South Africa

**Keywords:** Secondhand smoke, exposure, tobacco control

## Abstract

**Introduction:**

annually, many people die due to being exposed to secondhand smoke (SHS) which they experience at a number of premises that include health institutions. Scientists agree that there is no safe exposure level to SHS, however, in the City of Bulawayo many people are exposed to SHS. There are great expectations from communities for health professionals to reduce SHS exposure-related morbidity and mortality. This study sought to establish exposure to SHS in health institutions and sources of knowledge on SHS.

**Methods:**

a cross-sectional study, with participants randomly selected from residents visiting the 13 municipal revenue halls in the City of Bulawayo, was conducted. Data were collected through a structured questionnaire and were analyzed by performing descriptive and multivariate logistic regression.

**Results:**

26.3% (N = 419) of respondents who had been to health institutions in the previous 30 days had been exposed to SHS within those institutions. Almost all the respondents (85.4%) had never received a lesson on SHS from a health professional or had last received one three months before the survey. Furthermore, 74% of respondents had not seen posters on SHS or had last seen one more than three months before the survey.

**Conclusion:**

health professional should collaborate with other professionals in the fight against exposure to SHS as evidenced by the high prevalence of exposure in the health institutions and low health education given on SHS exposure in the City of Bulawayo

## Introduction

Exposure to secondhand smoke (SHS), also called unintentional smoking, leads to increased morbidity and mortality worldwide [1]. It is disturbing to note that many people suffer and some die due to unintentional smoking. Exposure to SHS occurs when tobacco smoke released during intentional smoking contaminates the air that non-smokers breath [1, 2]. SHS has over 4000 chemicals, with nearly 70 being cancer-causing agents [2, 3]. As such, exposure to SHS is linked with cancer, cardiac and lung diseases in grown-ups, and in children, breathing, ear infections, sudden infant syndrome and intellectual impairment [2,4,5]. SHS has no safe level of exposure [6]. All health institutions in Zimbabwe have been declared smoke free zone in terms of the National Tobacco Control Regulations [7]. These regulations envisage health institutions as places where people get health, not diseases or deaths. Tobacco surveys in India [8], Bangladesh [9] and Nigeria [10] show a low prevalence of exposure to SHS in health institutions (ranging between 5-6%) whereas in Egypt they show a seemingly high prevalence with 49.2% respondents in a Global Adult Tobacco Survey reporting exposure to SHS at a health institution in the 30 days prior the survey [11]. Health professionals, working together with environmental health specialists, town planning officers and law enforcement officers, should play a prominent role in the control of exposure to SHS within health institutions and in other public places. In Zimbabwe, health professionals such as environmental health practitioners are obliged, in terms of the law, to enter premises and enforce tobacco control legislation. Other health professionals such as medical doctors, health promotion officers and nurses, have numerous opportunities to engage the community in health promoting behaviors which include tobacco control. Health professionals provide an important interface with the community. The community, media and opinion leaders usually trust health professionals as they could convey messages on exposure to SHS across a vast range of social, economic and political arenas [12]. As health professionals interact with their clients, they can educate them on the harms of exposure to SHS and to all forms of tobacco use. These encounters between health professionals and their clients provide opportunities for the promotion of tobacco control actions from prevention of exposure to SHS to smoking cessation [13]. At community level, health professionals can support and implement provisions of tobacco control legislation to make public spaces such as health institutions, schools and public halls smoke free areas [12].

In spite of acceptance of the role of health professionals to advise clients on behavior changes to improve health [13], studies done in developing countries indicate that some health professionals are not making use of opportunities to advance the cause for tobacco control. One study established that in more than 95% of pediatric visits to health professionals, there was no discussion of tobacco control matters even where complaints linked to exposure to SHS were reasons for the visits [14, 15]. Some of the reasons for failure to take active roles in tobacco control activities included the use of tobacco products by the health professionals [12], lack of time [13, 15] or inadequate knowledge or confidence on tobacco control strategies [14] and anxiety to push away clients [16]. According to the World Health Organization, some programs of study for health professionals lack the right content and practice on smoking prevention and cessation and policy on tobacco control. This suggests that much effort is needed to make health professionals accept tobacco control as their responsibility [12]. Studies on the part played by health professionals in tobacco control largely report the perspectives of health professionals. Consequently, these studies have failed to capture the impact of health professionals’ practices on exposure to SHS in the community and the role of other health professionals such as environmental health practitioners and health promotion officers in tobacco control. Furthermore, there has been a small number of studies on the part played by health professionals in tobacco control activities in low to middle income countries as almost all studies focused on high income countries. Due to differences in availability of resources for tobacco control and the differences in the health professionals’ skills and attitudes towards tobacco control between high and low income countries, studies done in high income countries might fail to explain the situation prevailing in countries such as Zimbabwe. This present study, therefore, sought to find out exposure to and sources of knowledge on secondhand smoking in health institutions in the City of Bulawayo, Zimbabwe.

## Methods

**Study design and population:** a cross-sectional descriptive study was conducted among non-smoking adults in the City of Bulawayo. The 2012 census indicated the population in the City of Bulawayo as 303 346 males and 349 991 females [17]. Amongst these, 18.1% of the males and 0.7% of the females were classified as intentional smokers [18]. The distribution of the municipal housing offices in the City of Bulawayo gave rise to 13 sampling units. Adult residents, who are representative of the city population, visit these housing offices daily. A self-administered questionnaire was used to collect data on exposure to SHS in health institutions and sources of knowledge from non-smokers who consented to be part of the study. Only non-smoking residents above the age of 18 were qualified to take part in the study. Residents who quit intentional smoking in the last 6 months and those who were not intentionally smoking were considered as non-smokers. Smokers were considered to be those who were, at time of data collection, intentionally smoking tobacco.

**Sample size and sampling technique:** a minimum sample size of 399 (approximated to 400) was calculated using Yamane formula [19] based on a sampling error of 5% and population size of 653 337 in the City of Bulawayo [18]. Participants were sampled from the housing offices (n = 13) using systematic random sampling where every third person visiting a housing office was selected if meeting the inclusion criteria.

**Measures:** exposure to SHS was measured using the questions “In the last 30 days did you smell tobacco in a clinic, hospital or doctor’s private consulting rooms you visited?”, “Can a person be arrested for smoking tobacco in public places?”, Sources of information on SHS was measured using the questions “When was the last time you heard a health professional giving a lesson on the dangers of tobacco smoke to non-smokers?”; “When was the last time you read or saw a pamphlet or poster on the dangers of tobacco smoke to non-smokers?” The last two questions above were reformulated to give one dichotomous variable which had “active health education influence” and “inactive health education influence” as options. An active health education influence was considered as having heard health professionals giving lessons on exposure SHS or having seen health posters or messages in a period of 30 days or less prior to the survey, whereas having heard health professionals giving lessons on exposure SHS or having seen health posters or messages in a period of more than 30 days prior to the survey was considered as inactive health education influence.

**Ethical considerations:** the Research Ethics Committee of the University of Limpopo gave ethical clearance (MREC/HS/272/2014: PG) for the study while the City of Bulawayo approved the collection of data from participants visiting the various housing offices. Participants were requested to sign a consent form, which was in English and isiNdebele, to show their willingness to take part. Research ethics principles were respected and the questionnaire was translated into isiNdebele to give participants a choice of language.

**Data analysis:** to analyze data, SPSS 17.0 for windows version was used. Categorical and continuous data were summarized using proportions and mean±standard deviation, respectively. To compare the two groups, a Chi-square test and/or Fisher Exact test was used. A p-value of less than 0.05 was regarded as statistically significant.

## Results

**Demographic characteristics:** four hundred and twenty-six adults were approached of which 98% (n = 419) agreed to participate in the study. Slightly more than half (52%) of the respondents were females, nearly half (49%) were aged less than 35 years and nearly 47% had secondary school education. Fifty-two percent were married and/or living with a partner (Table 1).

**Table 1:** Socio-demographic characteristics of respondents (n=419)

	No	%
**Gender**		
Female	219	52
Male	200	48
**Age**		
18-24	97	23
25-34	111	26
35-44	75	18
45-54	63	15
55-64	57	14
65+	16	4
**Level of education**		
Primary	60	14
Secondary	197	47
Tertiary	162	39
**Marital Status**		
Single	133	32
Married/living with partner	218	52
Widowed	41	10
Divorced	26	6

**Prevalence and demographic factors associated with exposure to SHS:** sixty-four percent (270/419) of the respondents visited health institutions in the last 30 days before the survey. Of these, 26% (71/270) reported having smelled tobacco smoke in the health institutions they visited. Table 2 presents the relationship between exposure to SHS, gender and age for participants who visited health institutions in the 30 days before the survey. Males and respondents in the age group 35-54 years were more likely to report exposure to SHS than their counterparts, however, the results were not statistically significant (p>0.05).

**Table 2:** Association between exposure to SHS, gender and age

	N	Smelled tobacco smoke in a health premise in the last 30 days (%)		p-values
		Yes (n=71)	No (n=199)	
**Gender**				
Male	132	38(29)	94(71)	0.3630
Female	138	33(24)	105(76)	
**Age**				
<35	134	32(24)	102(78)	0.6680
35-54	92	26(30)	64(70)	
≥55	46	13(28)	33(72)	

**Figure 1:**
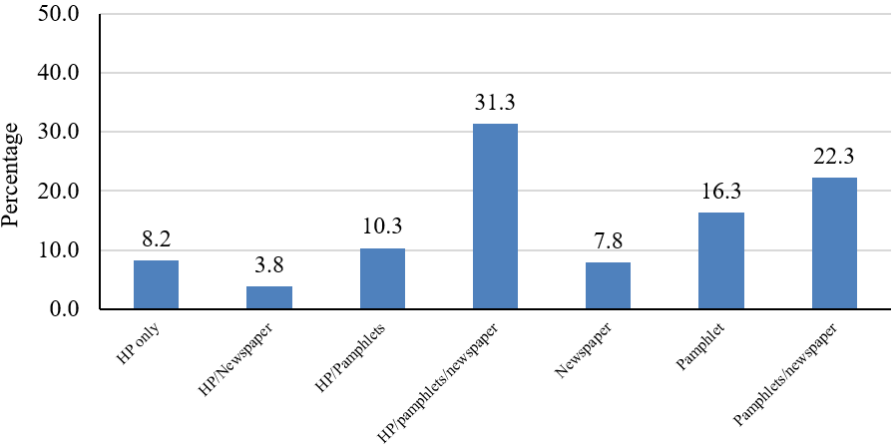
A) Line graph of the LVEF of IHD patients with and without HF across the four age groups; B) Kaplan Meier plot showing the risk of incident HF over 24-month period for an index IHD between 2015 and 2017

**Sources of information on exposure to secondhand smoke:** overall, 76% (319/419) of the participants indicated hearing or reading about dangers of exposure to SHS. Of these, 8% (n = 26) got information from health professional only and 45% (n = 145) got it from health professionals and other sources. There were 46% (n = 148) of the respondents who got information from pamphlets and newspapers. Detailed sources of information about the dangers of exposure to SHS are shown in Figure 1.

## Discussion

People visit health institutions to maintain their health and not to be exposed to hazards such as SHS. Pursuant to this expectation, the tobacco control legislation in Zimbabwe prohibits people to smoke within health institutions. Although there are various smells in the health institutions, however, this study discovered that more than a quarter (26%) of participants who visited health institutions claimed to have smelled tobacco thus exposed to it. The results of this study were considerably higher when compared to the prevalence of exposure to SHS in health institutions in developing countries such as India [8], Bangladesh [9], and Nigeria [10] where prevalence ranged between 5-6% in the preceding 30 days. However, the findings of this study were lower than the 49.2% reported in Egypt [11]. Health professionals are the custodians of the practices occurring within health institutions. Furthermore, health professionals such as environmental health practitioners are mandated by the legislation in Zimbabwe to enter any premise to monitor adherence to tobacco control regulations. Others, such as environmental health specialists, town planning officers and law enforcement officers, also have a responsibility to control exposure to SHS in public places. The high prevalence of exposure to SHS in health institutions within the City of Bulawayo might be suggestive of the fact that health professionals, working alone, fail to curb intentional smoking within health institutions. This claim is supported by the fact that many participants were not aware that smoking intentionally within health institutions was an offence a person can be arrested for. The study further revealed that health professionals were not actively involved in educating community members on tobacco control issues through giving health education to their clients frequently. The behavior of some health professionals in the City of Bulawayo was consistent with what has been reported elsewhere that health professionals seem reluctant to talk about tobacco control matters with their clients [14, 15]. Posters and pamphlets can complement the role of health professionals as they have an advantage of reaching where a health professional would not reach in person. They are even ideal in a cases where health professionals might be anxious to talk to patients about their smoking practices. Without distributing posters and fliers, it would be difficult for health professionals to penetrate the community with tobacco control messages [20].

When the two questions on health education given directly by health professionals and through pamphlets were combined into a single dichotomous variable, it was revealed that almost none of the participants had been subjected to active health education on the dangers of exposure to SHS. Many people might be exposed to diseases and deaths related to exposure to SHS as a result of access to sources of information that raises awareness about its dangers. It is worth noting that health professionals are held in high esteem in the communities where they work and have the potential to spread the message across a vast range of social, economic and political spheres [12]. Health professionals have an opportunity to educate clients on exposure to SHS during the client’s consultation [13]. Health promotion officers can utilize public health education sessions and as environmental health practitioners conduct regulatory visits to public facilities, they can enforce tobacco control legislation or educate the public on exposure to SHS. As previously reported, a significant portion of health professionals had inadequate knowledge and were not confident to advice on tobacco control, thus training health professionals in low to middle income countries as typified by the City of Bulawayo, Zimbabwe, is crucial in the fight against exposure to SHS. It is important, of course, to acknowledge that environmental health specialists, town planning officers and law enforcement offices also have a responsibility to control exposure to SHS in public places, thus the need for collaboration.

**Study limitation:** the limitation of this study is that the prevalence result is based on adult self-report and not on biomarker such as a cotinine. In addition, this study did not assess the social desirability effect as a source of error.

## Conclusion

A multidisciplinary team approach in the City of Bulawayo should do enough to control exposure to SHS as evidenced by the high prevalence of exposure to SHS in health institutions and the low health education given in the City of Bulawayo. A significant proportion of the non-smoking population in the City of Bulawayo was being exposed to SHS within health institutions despite the legislated and scientifically well-documented need for zero exposure to SHS. There is a need for training on tobacco control legislations and on the current tobacco control strategies so that non-smoking people are protect from the detrimental effects of exposure to SHS. Furthermore, health professionals should collaborate with other stakeholders such as environmental health specialists, town planning officers and law enforcement officers as they all have a responsibility to protect citizens from being exposed to SHS.

### What is known about this topic

- Secondhand smoke has no safe level of exposure;
- Health professional have a role to play in prevention of exposure to secondhand smoke;
- Environmental health specialists, town planning officers and law enforcement offices also have a role to play in prevention of exposure to secondhand smoke.

### What this study adds

- The study highlights that there is a high prevalence of exposure to secondhand smoke in health institutions in Zimbabwe, a developing country;
- Evidence for the need to train relevant stakeholders in developing countries on tobacco control activities in general and exposure to secondhand smoke in particular, as there is a need for them to educate communities on the dangers of exposure to secondhand smoke;
- The need for collaboration between health professionals and stakeholders such as environmental health specialists, town planning officers and law enforcement officers on prevention of exposure to secondhand smoke.

## Competing interests

The author declares no competing interests.
